# Scanning Electrochemical Microscopy of Single-Crystal Platinum Electrode

**DOI:** 10.1021/acs.analchem.5c07593

**Published:** 2026-01-22

**Authors:** Donald C. Janda, George W. Fritze, Ryan D. Tate, William Strang, Nagahiro Hoshi, Shigeru Amemiya

**Affiliations:** † Department of Chemistry, 6614 University of Pittsburgh, Pittsburgh, Pennsylvania 15260, United States; ‡ Department of Applied Chemistry and Biotechnology, Graduate School of Engineering, 12737 Chiba University, Chiba 263-8522, Japan

## Abstract

The surface of single-crystal metal electrodes can be well controlled at the atomic level to serve as superior models for fundamental electrochemistry in comparison with the polycrystalline counterparts. The single-crystal surface of a metal can be cleaned thermally and oriented downward to form a meniscus contact with an electrolyte solution for diverse electroanalytical characterization. Problematically, the widely used hanging-meniscus configuration is incompatible with scanning electrochemical microscopy (SECM), which requires the upward orientation of the single-crystal surface to an ultramicroelectrode tip. Herein, we report a precision-made glass cell to enable SECM of a disk Pt(111) substrate as a well-established model of single-crystal metal electrodes. The clean glass cell can accommodate the flame-annealed Pt(111) disk without adventitious contamination and solution leakage. The cleanliness of the entire Pt(111) surface is confirmed by cyclic voltammetry in perchloric acid and sulfuric acid to observe characteristic surface waves with butterfly peaks. We employ SECM to monitor the redox dynamics of underpotential hydrogen deposition, hydroxyl adsorption, and hydrogen oxidation at the clean Pt(111) surface under the tip. These electron-transfer reactions are coupled with proton transfer to generate and consume H^+^, which is detected amperometrically at the tip while the substrate potential is cycled. Interestingly, the tip current changes only slightly while a sharp butterfly peak is observed at the substrate, thereby indicating an unexpected nonfaradaic origin of the well-known peak. The new glass cell will be useful for in situ SECM of electrocatalytic reactions and intermediates at various single-crystal metal electrodes.

## Graphical abstract

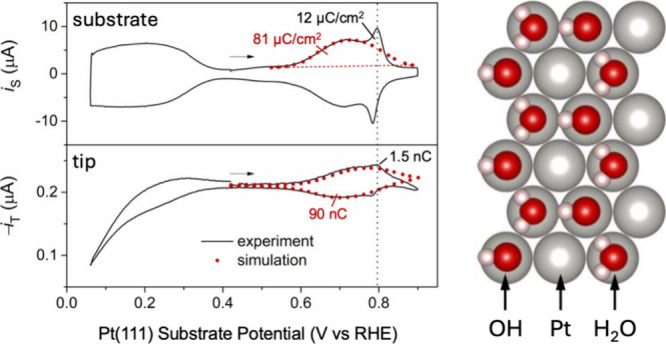

A wide range of electroanalytical methods has been applied to investigate atomically well-defined surfaces of single-crystal metal electrodes for fundamental electrochemistry.^1^ Cyclic voltammetry (CV) of single-crystal Pt electrodes revealed the facet-dependent electrodeposition of hydrogen, H_ads_, from H^+^ through the Volmer reaction^2^

$$H^{+}+e⇌H_{\mathrm{ads}}$$ 1

Characteristic surface waves are observed at more positive potentials than the hydrogen evolution reaction owing to the strong interaction of the Pt surface with underpotential deposited hydrogen. Moreover, the Pt(111) surface yields butterfly like surface waves with sharp and broad peaks,^3,4^ which have been attributed to the formation of hydroxyl adsorbate, OH_ads_, through^5^

$${\mathrm{OH}}_{\mathrm{ads}}+H^{+}+e⇌H_{2}O$$ 2

Importantly, the single-crystal surface must be clean to quantitatively observe the surface waves of H_ads_ and OH_ads_. Experimentally, the single-crystal surface of a metal can be cleaned thermally and oriented downward to form a meniscus contact with an ultrapure aqueous electrolyte for electroanalytical characterization.^6^ The hanging-meniscus configuration is compatible even with rotating-electrode voltammetry.^7^

Herein, we report a precision-made glass cell (Figure 1 and Figure S-1) to enable the reliable in situ study of single-crystal metal electrodes by scanning electrochemical microscopy^8–10^ (SECM). Significantly, we find that the sharp butterfly peak of a voltammogram at the Pt(111) surface is nonfaradaic^11^ in contrast to the traditional faradaic view (eq 2).^5^ This new information was obtained uniquely by employing SECM, which can selectively measure the consumption and generation of H^+^ during faradaic hydroxyl adsorption against nonfaradaic double-layer charging. Current responses based on faradaic adsorption and nonfaradaic charging are coupled in other electrochemical methods as represented by CV.^11,12^ Previously, neither underpotential hydrogen deposition nor hydroxyl adsorption was investigated by SECM of single-crystal Pt surfaces.^13,14^ Moreover, an SECM cell was assembled in a vacuum to maintain a clean Pt(111) surface.^14^ By contrast, our glass cell can be assembled without a vacuum to orient a flame-annealed Pt(111) surface upward to an ultramicroelectrode tip. The cleanliness of the Pt(111) surface is confirmed by characteristic surface waves as observed not only entirely by CV but also locally by SECM.

**Figure 1:**
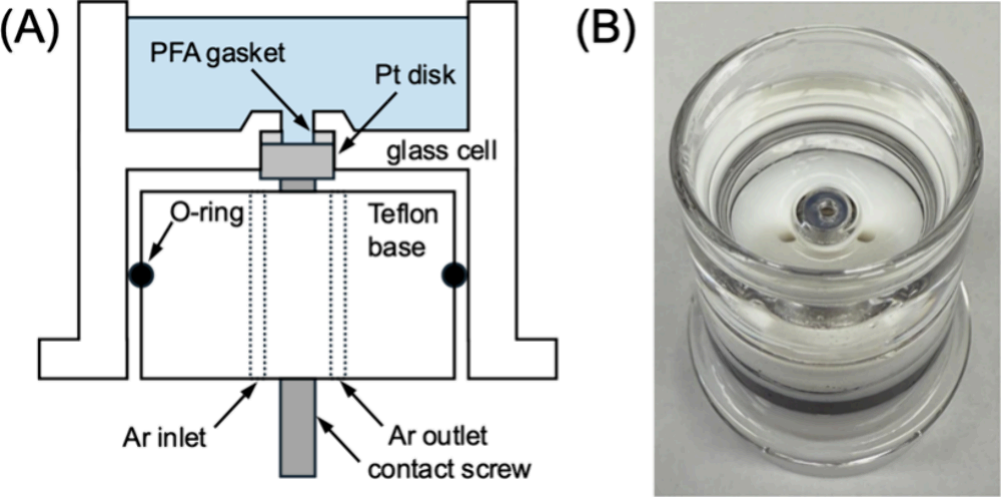
(A) Scheme and (B) photo of a glass cell with a Pt(111) disk electrode.

Specifically, our glass cell was assembled within a few minutes in a benchtop clean hood without adventitious contamination and solution leakage (Figure 2). Cell components were cleaned in hot sulfuric acid, sonicated in and rinsed with ultrapure water^15^ as detailed in Supporting Information (SI). A wet clean glass cell was flipped and soaked in the target electrolyte of ultrapure water (Figure 2A). A 0.5 mm-thick PFA gasket with a ∼3 mm-diameter hole was also cleaned and placed on the precisely made well of the glass cell (Figure 2B). The level of the electrolyte was set just above the gasket by removing the extra solution from the exterior of the glass cell. A flame-annealed Pt(111) disk was protected by a droplet of ultrapure water and placed downward on the PFA gasket to fit the glass well (Figure 2C). The extra electrolyte around the Pt disk was removed until the resultant solution level in the well was set slightly above the Pt(111) surface. A Teflon base and a contact screw were attached to the glass cell while high-purity Ar was purged from behind (Figure 2D). The assembled glass cell and glass container were flipped together to orient the Pt(111) surface upward (Figure 2E). The cell was sandwiched between two metal plates by screws^16^ and mounted on goniometer stages (Figure S-2A). The screws tightened the contact between the PFA gasket and the ripples formed around the ∼3 mm-diameter opening of the glass well (Figure 2F) to prevent the solution leakage (Figure S-2B). A glass cell without the ripples leaked to contaminate the Pt(111) surface. The electrolyte was purged with high-purity Ar for 10 min to remove O_2_ before electrochemical measurements.

**Figure 2:**
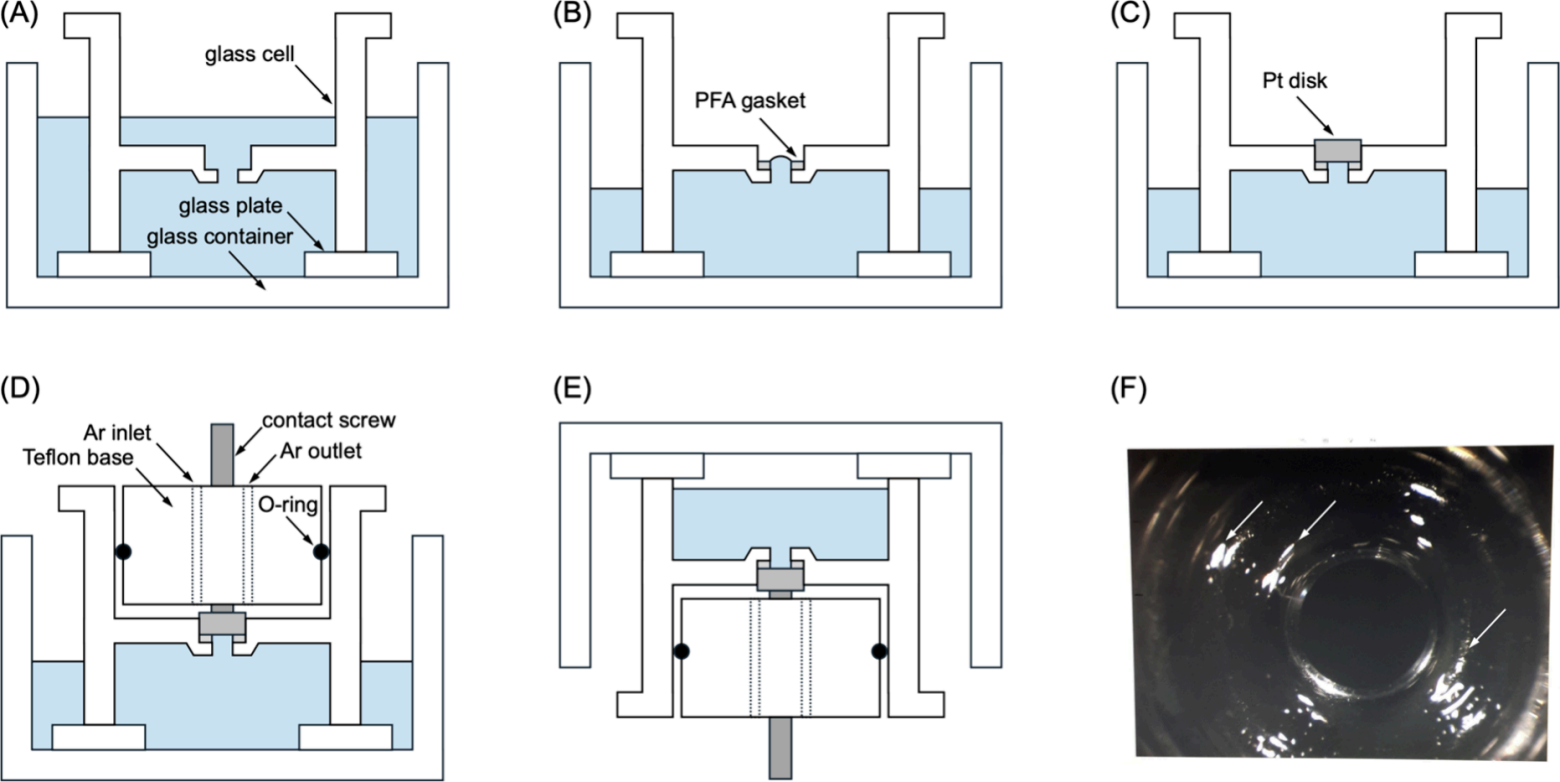
(A)–(E) Assembly of a glass cell with a flame-annealed Pt disk electrode. (F) A photo of ripples formed around the opening of a glass cell. Arrows indicate white reflections from ripples around the opening.

The cleanliness of the Pt(111) surface in the glass cell was quantitatively confirmed by characteristic voltammetric features of hydrogen and hydroxyl adsorption in 0.1 M HClO_4_ (black line in Figure 3A). The surface wave of underpotential hydrogen deposition (eq 1) was observed at <0.35 V and broadened by strong repulsion among H_ads_.^17^ The butterfly like surface wave of hydroxyl adsorption (eq 2) at ∼0.65 V was also broadened by strong repulsion among OH_ads_ and followed by a sharp peak at ∼0.8 V.^18^ We performed the microkinetic analysis of substrate voltammograms to determine relevant reaction parameters, which are consistent with those reported in the literature.^17,18^ A good fit of underpotential hydrogen deposition (blue dots in Figure 3A) yielded the saturated surface concentration of 2.5 nmol/cm^2^ (blue line in Figure 3B). This value is equivalent to the surface concentration of Pt atoms on the (111) facet,^1^ thereby indicating that three H_ads_ atoms are adsorbed on the three hollow sites of one Pt(111) atom.^19^ In addition, the fit yielded a formal potential, *E*_V_^0^′^^, of 0.295 V and a H_ads_–H_ads_ repulsion parameter, *g*_H_^′^, of −11. A good fit of the broad butterfly peak (red dots in Figure 3A) gave a formal potential, *E*_OH_^0^′^^, of 0.67 V and an OH_ads_–OH_ads_ repulsion parameter, *g*_OH_^′^, of −4.5. Moreover, the saturated charge of 79 μC/cm^2^ was determined for the surface OH_ads_ concentration of 0.83 nmol/cm^2^ (red line in Figure 3B). This result indicates that one OH_ads_ is formed on one of three Pt atoms as predicted by a DFT model,^20^ which cannot account for the sharp butterfly peaks. The total charge including the sharp butterfly peak is larger by 22% than that of the broad peak to reach 96 μC/cm^2^ (Figure 3A).

**Figure 3:**
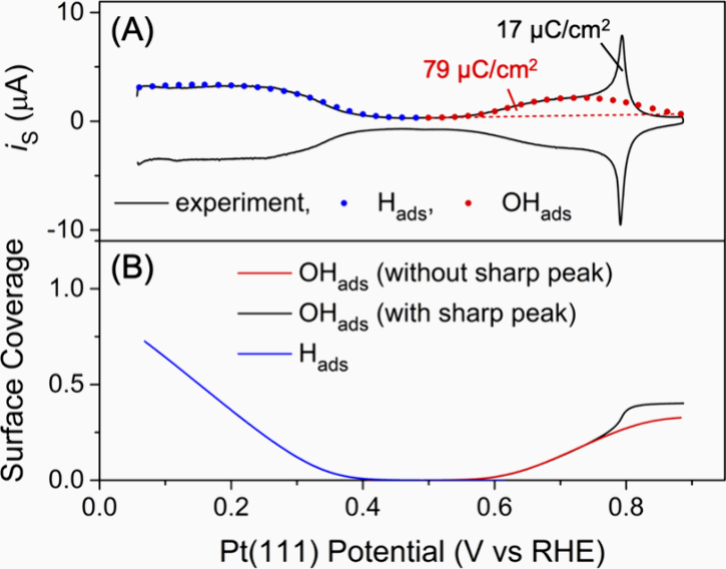
(A) CV of a Pt(111) disk electrode at 0.05 V/s in 0.1 M HClO_4_ in the glass cell. Red and blue dots represent the simulation. (B) Simulated surface coverages of H_ads_ and OH_ads_ (blue and red lines, respectively). The black line includes the surface coverage that corresponds to the charge of the sharp butterfly peak.

We applied the adsorption-coupled electron-transfer mode^21^ of SECM (Figure 4A) to measure the consumption and generation of H^+^ through hydrogen and hydroxyl adsorption on the Pt(111) substrate. We employed a 25 μm-diameter Pt tip, which was large enough to observe a transient tip current response to hydrogen adsorption at the polycrystalline Pt substrate.^22^ The tip reduced H^+^ to H_2_ at −0.2 V to yield a diffusion-limited current response, *i*_T_, without contributions of hydrogen or hydroxyl adsorption. A low concentration of 0.5 mM HClO_4_ was used to avoid the formation of H_2_ bubbles,^23^ which can interfere with the tip and substrate reactions. The tip was positioned at a few micrometers from the Pt(111) surface by measuring an approach curve^24^ (Figure S-3). The potential of the Pt(111) substrate was initially set at 0.4 V and cycled at 0.1 V/s to observe characteristic current responses, *i*_S_, at the substrate (Figure 4B). A sharp butterfly peak in 0.5 mM HClO_4_ and 0.1 M KClO_4_ was lower and broader than in 0.1 M HClO_4_ (Figure 3A) but substantial.

**Figure 4:**
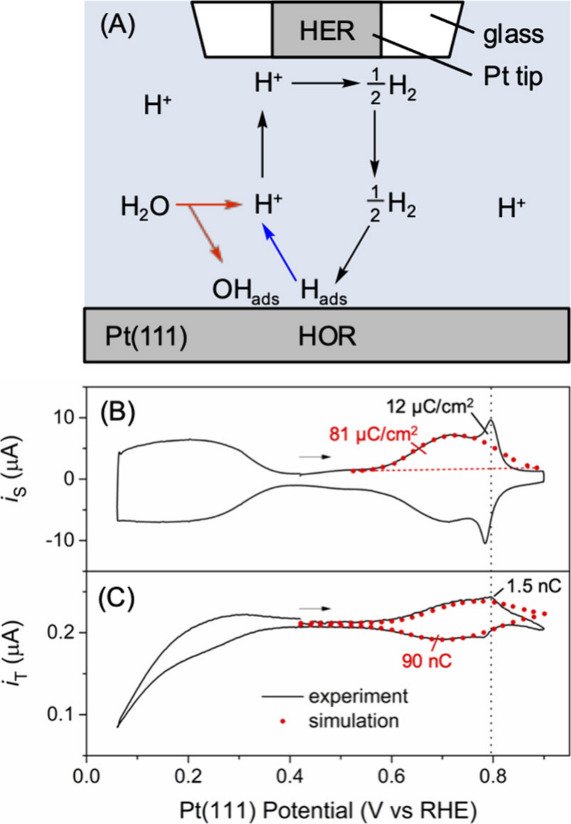
(A) Adsorption-coupled electron-transfer mode of SECM based on hydrogen evolution and oxidation reactions at the tip and the substrate, respectively. (B) Substrate and (C) tip current responses against the potential of the Pt(111) substrate at 0.10 V/s in the Ar-purged ultrapure-water solution of 0.5 mM HClO_4_ and 0.1 M KClO_4_. The tip–substrate distance, 2.3 μm.

The hysteresis of the tip current was observed (Figure 4C) simultaneously with broad peaks of hydrogen and hydroxyl adsorption at the substrate (Figure 4B). As the substrate potential was scanned initially from 0.4 to 0.8 V, hydroxyl adsorption on the Pt(111) surface transiently generated H^+^ (eq 2), which increased the concentration of H^+^ near the tip. The resultant tip current was enhanced to yield a broad peak. Interestingly, the tip current showed only a very small peak at ∼0.8 V (dotted line), where a sharp butterfly peak was observed at the Pt(111) substrate. The hysteresis of the tip current was enhanced with shorter tip–substrate distances of down to 0.68 μm and faster scan rates of up to 1 V/s. By contrast, the peak current response of the tip at ∼0.8 V remained small. The small peak was not due to the contamination of the substrate by the tip and was unchanged when the tip was cleaned by a piranha solution or a flame. The small peak at the tip indicates that H^+^ was generated and consumed much less than expected from the sharp peak at the substrate as discussed more quantitatively below. A broad hysteresis of the tip current was observed for hydrogen adsorption on the Pt(111) surface,^19^ where H^+^ was consumed and generated as the substrate potential was scanned at <0.35 V.

We performed the microkinetic analysis of SECM-based voltammograms^19^ (also see SI) to simulate the tip current based on the hydrogen evolution reaction (Figure 4A). The tip reaction was diffusionally coupled with the hydrogen oxidation reaction at the substrate under the tip. The simulated current agreed very well with the experimental one at the negative side of the sharp butterfly peak when hydroxyl adsorption on the substrate was also considered (Figure 4C). Both experimental and simulated tip currents were steady while the substrate current increased to top the sharp butterfly peak at ∼0.8 V (Figure 4B). The experimental tip current decreased at >0.8 V to go below the simulated tip current. This result is consistent with the transition of the hydroxyl adlayer from a disordered state to an ordered state at ∼0.8 V.^20^ The ordered adlayer is dense enough to hinder the hydrogen oxidation reaction at the substrate under the tip (Figure 4A), thereby producing fewer H^+^ to lower the tip current. The tip current was also lowered at <0.2 V, where the hydrogen oxidation reaction slowed down.^19^ We were not able to simulate the SECM-based voltammogram of underpotential hydrogen deposition without knowing the mechanism of the hydrogen oxidation reaction.^19^

Excellent fits between experimental and simulated tip currents at the negative side of the sharp butterfly peak provided quantitative insights into hydroxyl adsorption. The fits were obtained by adjusting only an OH_ads_–OH_ads_ repulsion parameter, *g*_OH_^′^, of −3.3 ± 0.2 (*N* = 8). We also determined the saturated surface concentration of 0.83 nmol/cm^2^ for OH_ads_ on the (111) surface with the Pt atom density, Γ_s_, of 2.5 nmol/cm^2^ as expected from the DFT-based prediction.^20^ Moreover, we obtained the formal potential, *E*_OH_^0^′^^, of 0.70 V and estimated a standard rate constant, *k*_OH_^0^, of 4.0 s^–1^ as the diffusion-limited minimum value. The parameters determined by SECM were used to simulate the corresponding substrate CV (red dots in Figure 4B), which fitted well with the experimental one. The good fit, however, required the subtraction of the background charging current, which was absent in the amperometric tip current (Figure 4C) as an advantage of SECM over CV.^25^

We also used the microkinetic analysis to quantitatively demonstrate that H^+^ was generated and consumed much less than expected from the sharp butterfly peak at ∼0.8 V. A charge of 90 nC is surrounded by forward and reverse waves of the simulated SECM voltammogram (red dots in Figure 4C). This charge corresponds to the total charge of H^+^ generated and consumed by hydroxyl adsorption and desorption at the substrate (eq 2), respectively. We estimate that half of the total charge (45 nC) is attributed to the charge based on hydroxyl adsorption. The charge of the small peak above the simulated voltammogram at ∼0.8 V is 1.5 nC, which is equivalent to only 3.3% of the charge associated with hydroxyl adsorption. By contrast, the charge of the sharp butterfly peak above the simulated substrate CV in the same electrolyte corresponds to ∼15% of the charge under the background-corrected broad peak (12 and 81 μC/cm^2^, respectively, in Figure 4B). These results indicate that a large fraction of the charge based on the sharp butterfly peak as observed at the substrate does not involve the generation or consumption of H^+^ through hydroxyl adsorption.

We propose that the sharp butterfly peak is mainly attributed to the nonfaradaic current^11^ based on the adsorption of perchlorate on the hydroxyl adlayer without the generation or consumption of H^+^. Raman spectroscopy demonstrated that perchlorate is adsorbed favorably on the ordered hydroxyl adlayer in comparison with the disordered one.^26^ The abrupt order–disorder transition at ∼0.8 V can induce the nonfaradaic adsorption and desorption of perchlorate as a carrier of charging current to generate the sharp peak. It should be noted that perchlorate is not oxidatively^4,20^ or specifically^26^ adsorbed on the Pt(111) surface. A sharp nonfaradaic peak based on the order–disorder transition of an adlayer was also reported for the coadsorption of diphenyl viologen with chloride as a carrier of charging current on the Au(111) electrode.^27^ Moreover, we attribute small peaks at ∼0.8 V in SECM-based voltammograms to the enhancement of hydroxyl adsorption by the phase transition of the adlayer^28^ or the adsorption of the second hydroxyl species.^18^ Previously, these two faradaic mechanisms were proposed as the sole origins of sharp butterfly peaks.

We also applied SECM to investigate butterfly peaks as observed by CV of the clean Pt(111) surface in 1.0 mM H_2_SO_4_ and 0.1 M KClO_4_ (Figure 5A). These butterfly peaks have also been observed in high concentrations of H_2_SO_4_ (0.5 M in Figure S-4) and attributed to the oxidative adsorption of sulfate^29^ as an inhibitor of the oxygen reduction reaction.^30^ We employed 1.0 mM H_2_SO_4_ to ensure that the H^+^ concentration near the tip was changed by hydrogen adsorption at <0.35 V to alter the tip current (Figure 5B). By contrast, we found that the tip current did not change during the occurrence of butterfly peaks (>0.5 V in Figure 5) at the Pt(111) substrate. The lack of the tip current response to the broad prepeak indicates that sulfate adsorption is mediated without proton transfer as reported previously.^31^ The corresponding reaction is given by

$${\mathrm{SO}}_{4\mathrm{ads}}^{-}+e^{-}⇌{\mathrm{SO}}_{4}^{2-}$$ 3

where SO_4ads_^–^ is the adsorbate. Our result excludes the adsorption of sulfate from bisulfate, which requires proton transfer as indicated by

$${\mathrm{SO}}_{4\mathrm{ads}}^{-}+H^{+}+e^{-}⇌{\mathrm{HSO}}_{4}^{-}$$ 4

**Figure 5:**
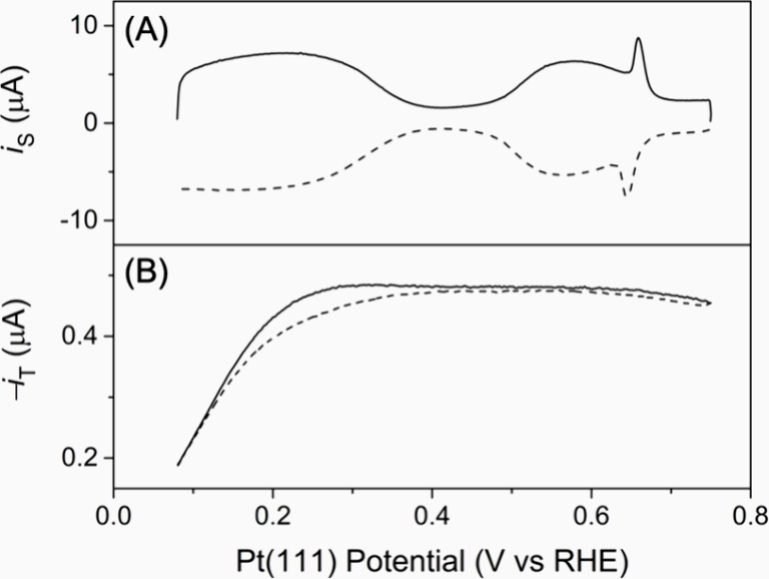
(A) Substrate and (B) tip current responses against the cycled potential of the Pt(111) substrate at 0.1 V/s in the Ar-purged ultrapure-water solution of 1.0 mM H_2_SO_4_ and 0.1 M KClO_4_. Solid and dashed lines represent forward and reverse scans, respectively. The tip–substrate distance, 2.4 μm.

The former reaction is thermodynamically preferred at pH ∼ 3, where sulfate is ∼10 times more abundant than bisulfate (p*K*_a_ = 2).^32^ Unfortunately, SECM cannot assess without proton transfer whether the sharp peak with the sulfate solution is faradaic or nonfaradaic. The latter, however, is supported by a change in the double-layer capacitance at the potential of the sharp butterfly peak,^33^ where the abrupt order–disorder phase transition was observed by STM.^34,35^ We propose that additional sulfate molecules are adsorbed on the ordered sulfate–water adlayer to serve as nonfaradaic current carriers for the sharp peak during the phase transition.

## Conclusions

In this work, we developed a precision-made glass cell to enable SECM of the clean Pt(111) disk electrode. The adsorption-coupled electron-transfer mode of SECM^21^ was employed to quantitatively observe underpotential hydrogen deposition and hydroxyl adsorption, thereby confirming the cleanliness of the local Pt(111) surface under the tip. Moreover, an SECM-based voltammogram was quantitatively analyzed without the CV of the entire substrate to independently determine the parameters of the local substrate reactions under the tip. The precision-made glass cell will be useful for SECM of electrocatalytic reactions at various single-crystal metal electrodes under high mass-transport conditions to provide new insights into reactive intermediates and reaction pathways.^19^ The power of the new electroanalytical capability was demonstrated by a finding that sharp butterfly peaks appeared much lower for SECM of the Pt(111) substrate than for CV. More work will be reported to assess whether our nonfaradaic mechanism can be modeled to quantitatively account for sharp butterfly peaks with not only perchlorate but also other anions.^36^

## Supplementary Material



## Notes

Chemicals and materials, glass cell, SECM measurement, and microkinetic analysis (PDF)
