# Supplementary material for: Scanning Electrochemical Microscopy of Single-Crystal Platinum Electrode
Source: Anal Chem. 2026 Jan 22;98(4):2686–91. doi: 10.1021/acs.analchem.5c07593 (PMC12874217; doi:10.1021/acs.analchem.5c07593)
Supplement: Supplementary file 1 [file ac5c07593_si_001.pdf]

Supporting Information

Scanning Electrochemical Microscopy of Single-Crystal Platinum Electrode

Donald C. Janda,[†] George W. Fritze,[†] Ryan D. Tate,[†] William Strang,[†] Nagahiro Hoshi,[‡] and Shigeru Amemiya[†,*]

[†] Department of Chemistry, University of Pittsburgh, Pennsylvania 15260, United States

[‡] Department of Applied Chemistry and Biotechnology, Graduate School of Engineering, Chiba University, Chiba 263-8522, Japan

* To whom correspondence should be addressed. E-mail: amemiya@pitt.edu. Fax: 412-624-8611.

Contents:

1. Chemicals and Materials S-2

2. Glass Cell S-2

3. SECM Measurement S-4

4. Microkinetic Analysis S-6

5. References S-14

Chemicals and Materials. $HClO_4$ (70%, Suprapur), H_2SO_4 (96%, Suprapur), and $KClO_4$ (EMSURE, 99.99% purity) were obtained from MilliporeSigma (Burlington, MA). $HClO_4$ (70%, ULTREX) was purchased from Avantor (Radnor, PA) to obtain cyclic voltammograms (CVs) of 0.1 M $HClO_4$. Single-crystal Pt(111) disks with 99.999% purity, 5.00 mm diameter, and 4.00 mm thickness as polished with <0.01 μm roughness and <0.1 degree orientation accuracy were purchased from Princeton Scientific (Easton, PA). A 0.5 mm-thick film of Teflon PFA copolymer was obtained from GoodFellow (Pittsburgh, PA). A Milli-Q IQ 7003 water purification system (EMD Millipore, Billerica, MA) was used to obtain UV-treated deionized ultrapure water (18.2 MΩ·cm) with total organic carbon of 4–5 ppb. High-purity Ar (99.9999%) and H_2 (99.999%) were obtained from West Penn Laco (Pittsburgh, PA) and Matheson (Irvin, TX), respectively.

Glass Cell. A glass cell was prepared from a Pylex tubing (ID 25 mm and OD 30 mm in Figure S-1A), cleaned, and assembled as follows. The end of the tube was closed and made into a flat bottom (Figure S-1B) to open a ~2 mm-diameter hole (Figure S-1C). The small hole was made by using a small jeweler's torch and spinning the glass tube mounted on the lathe. Specifically, the center of the flattened bottom was melted by the small flame and raised by applying air pressure immediately after the flame was removed. A glass rod was used to remove a small amount of glass from the face of the small protrusion while being struck by the flame. Air pressure was applied to pop the protrusion in the flame, thereby opening the small hole. A stainless-steel tool was made with a cylindrical protrusion to mimic the Pt(111) disk (Figure S-1D). The protrusion was pressed into the heated glass bottom to leave an open well to accommodate the disk (Figure S-1E). This process also increased the hole diameter to ~3 mm. Importantly, the tool was kept as cool as touchable by a bare hand to form ripples around the opening of the well bottom (Figure 2F). The cell was completed by sealing a tube to the flat bottom (Figure S-1F). A slight flare was made on the end of the bottom tubing to facilitate the insertion of the Teflon base.

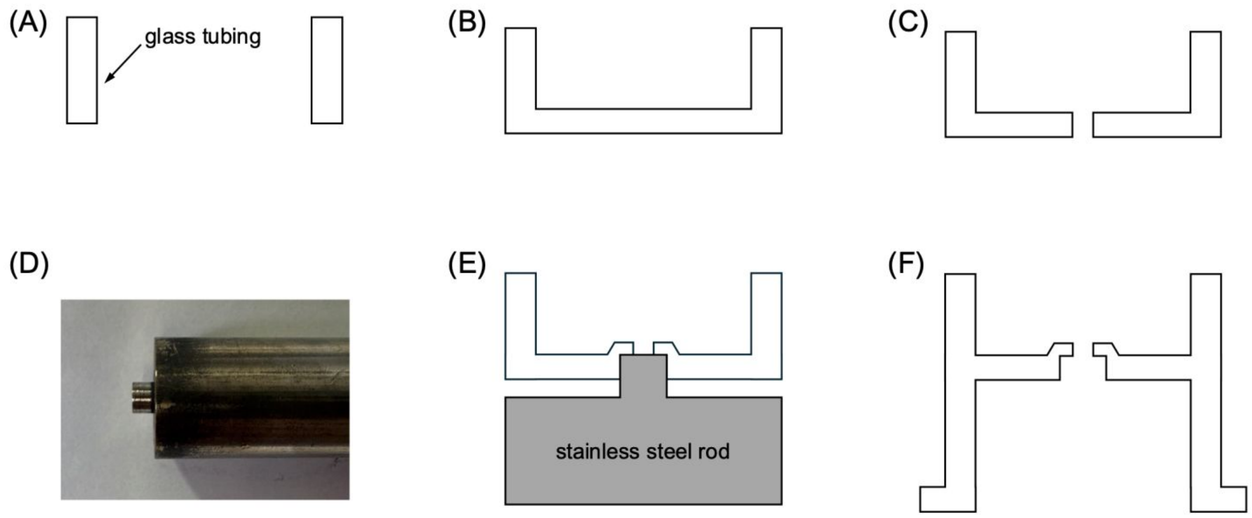

Figure S-1. Preparation of a glass SECM cell.

A glass cell was assembled in a class 100 vertical laminar flow hood (AC632TLFC, AirClean Systems, Raleigh, NC) equipped with a bonded carbon filter (ACF100, AirClean Systems). Cell components were made of glass or Teflon, cleaned in concentrated sulfuric acid at 80 °C for 60 minutes or 5 minutes, respectively, rinsed with ultrapure water, sonicated in ultrapure water for 30 minutes, and stored in ultrapure water for less than 2 hours before assembly. A Pt(111) disk was annealed in a H_2/air flame at ~1600 ºC for 5 minutes, cooled in Ar/H_2 (97:3) for 2.5 minutes, and stored in ultrapure water. The glass cell was assembled as detailed in the main text and mounted on goniometer stages (Figure S-2A). The glass cell was sandwiched between top and bottom metal plates by screws, which also tightened the contact between the PFA gasket and the ripples around the opening of the glass cell (Figure S-2B) to prevent solution leakage.

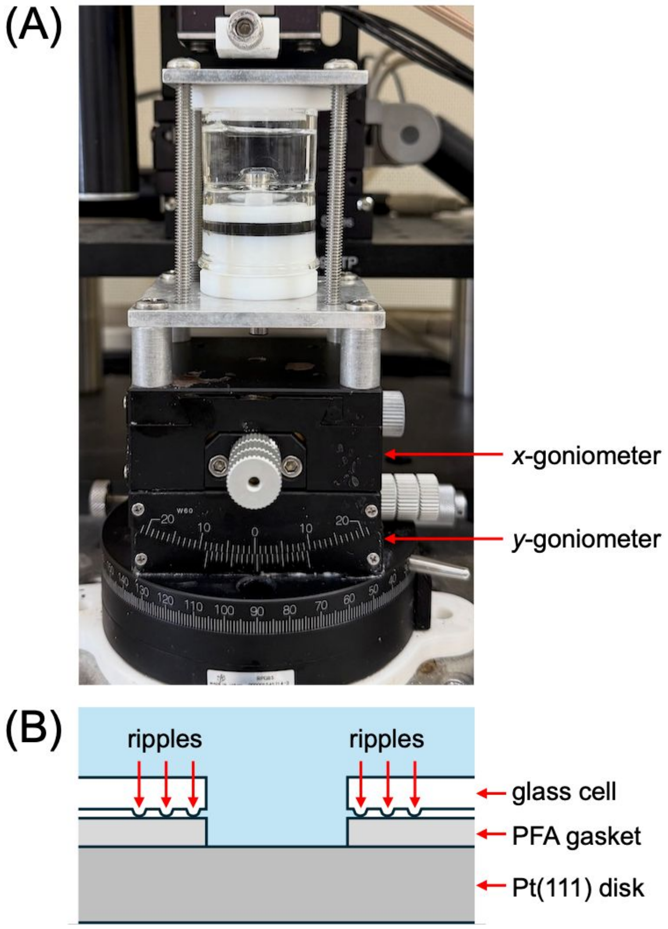

Figure S-2. (A) A photo of a glass cell mounted on goniometer stages. (B) Scheme of the contact between the PFA gasket and the ripples around the opening of the glass cell.

SECM Measurement. We employed a commercial SECM instrument (CHI 910B, CH Instruments, Austin, TX) to measure the amperometric response of a 25 µm-diameter Pt tip. The tip was sealed in a pulled glass capillary,[S-1] and milled by the focused Ga^+ beam (30 keV) using a dual-beam instrument (Scios, FEI, Hillsboro, OR). The Pt tip was cleaned in piranha solution (a 1:3 mixture of 30% H_2O_2 and 95.0–98.0% H_2SO_4) and ultrapure water immediately before the tip was immersed in the electrolyte solution of the cell. Caution: Piranha solution reacts violently with organics and should be

handled with extreme care! The tip was also cleaned in a H_2/air flame. The Pt tip was positioned near the Pt(111) surface by measuring an approach curve in the steady-state feedback mode (Figure S-3). The experimental approach curve agreed well with an analytical approximation of simulated approach curve[S-2] to ensure that hydrogen evolution and oxidation reactions at the tip and the substrate, respectively, are diffusion-limited. The diffusion-limited hydrogen evolution reaction was driven at the tip with a Pt quasi-reference/counter electrode by using the potentiostat of CHI 910B while the substrate potential was positive enough to drive the diffusion-limited hydrogen oxidation reaction.

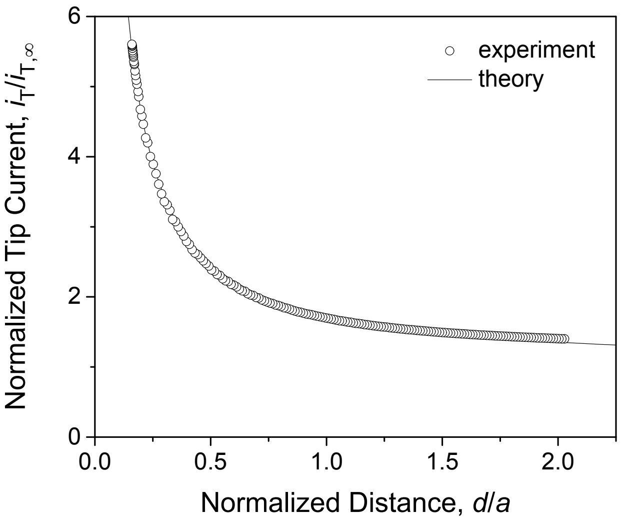

Figure S-3. Experimental and theoretical approach curves of a 25µm-diameter Pt tip at the Pt(111) disk in the Ar-purged ultrapure water solution of 0.5 mM $HClO_4$ and 0.1 M $KClO_4$. The tip currents near the Pt(111) surface and in the bulk solution are represented by i_T and $i_{T,\infty}$, respectively.

A floating potentiostat (CHI 660B, CH Instruments) was employed to cycle the potential of the Pt(111) substrate with a Pt quasi-reference electrode and a Pt counter electrode. The ohmic drop of the substrate potential was minimal with the scan rate of 0.1 V/s and compensated. The floating potentiostat was synchronized with the grounded potentiostat of CHI 910B but isolated from each other electronically by using a digital switch with an optical coupler.[S-3] The cleanliness of the Pt(111) surface was checked by CV with 0.1 M $HClO_4$ (Figure 3A) and 0.5 M H_2SO_4 (Figure S-4). Electrode potentials were calibrated against a reversible hydrogen electrode (MiniHydroFlex, Biologic, Knoxville, TN).

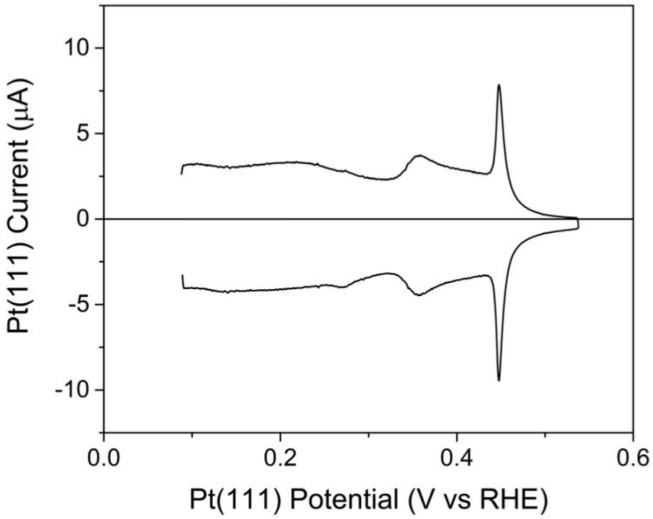

Figure S-4. CV of a Pt(111) disk electrode at 0.05 V/s in the Ar-purged ultrapure-water solution of 0.5 M H_2SO_4 in the glass cell.

Microkinetic Analysis. A diffusion–reaction model was developed for hydrogen adsorption and oxidation reactions in our recent work[S-4] and modified for the microkinetic analysis of hydroxyl adsorption in this work. The diffusion–reaction problem was solved by using COMSOL Multiphysics

(version 6.2, COMSOL, Burlington, MA) to calculate the Faradic current based on the diffusion-limited hydrogen evolution reaction at the tip, i_T. The simulated tip current was plotted against the substrate potential to fit experimental voltammograms (Figure 4C). Specifically, the diffusion problem was defined by setting up an SECM configuration in the cylindrical coordinates (Figure S-5). Time-dependent diffusion equations for H^+ and H_2 were given in cylindrical coordinates as

$$\frac{\partial c_{H^+}}{\partial t} = D_{H^+}\left(\frac{\partial^2 c_{H^+}}{\partial r^2} + \frac{1}{r}\frac{\partial c_{H^+}}{\partial r} + \frac{\partial^2 c_{H^+}}{\partial z^2}\right) \tag{S-1}$$

$$\frac{\partial c_{H_2}}{\partial t} = D_{H_2}\left(\frac{\partial^2 c_{H_2}}{\partial r^2} + \frac{1}{r}\frac{\partial c_{H_2}}{\partial r} + \frac{\partial^2 c_{H_2}}{\partial z^2}\right) \tag{S-2}$$

where c_i and D_i are the concentration and diffusion coefficient of a species i (= H^+ or H_2) in the solution.

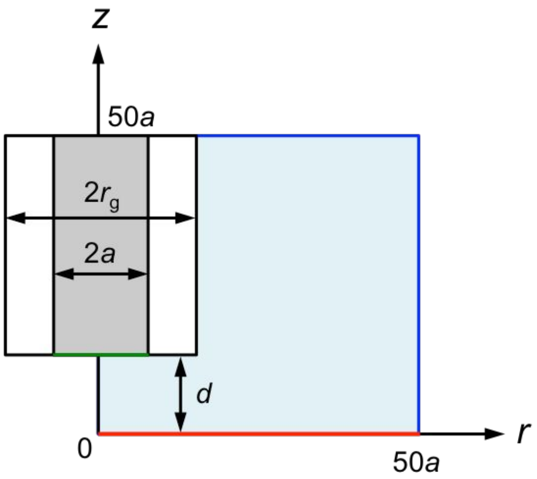

Figure S-5. Scheme of the SECM configuration with a glass-insulated Pt tip positioned over a macroscopic Pt substrate. The red boundary represents the substrate surface. The green boundary represents the tip surface. Black boundaries are insulating or a symmetry axis. Blue boundaries represent the bulk solution.

Boundary conditions at the tip and substrate surfaces are given as follows. The tip potential was set sufficiently negative to drive the diffusion-limited hydrogen evolution reaction (HER). The corresponding rate, v_{HER}, was defined by the one-step reaction of H^+ to H_2 without the consideration of H_{ads} as

$$v_{HER} = k_{HER}\left(c_{H^+}\right)^2 \tag{S-3}$$

where k_{HER} is the HER rate constant. Boundary conditions at the tip are given by

$$D_{H^+}\left(\frac{\partial c_{H^+}}{\partial z}\right) = -2v_{HER} \tag{S-4}$$

$$D_{H_2}\left(\frac{\partial c_{H_2}}{\partial z}\right) = v_{HER} \tag{S-5}$$

By contrast, the substrate mediated the diffusion-limited hydrogen oxidation reaction (HOR) as well as the transient hydroxyl adsorption. The rate of the diffusion-limited HOR, v_{HOR}, was given by

$$v_{HOR} = k_{HOR}c_{H_2} \tag{S-6}$$

where k_{HOR} is the HOR rate constant. The potential-dependent rate, v_{OH}, of hydroxyl adsorption was given by

$$v_{OH} = k_{OH}^{ads}\frac{c_{H^+}}{c_0}\Gamma_{OH} - k_{OH}^{des}\left(\Gamma_s - \Gamma_{OH}\right) \tag{S-7}$$

with

$$k_{OH}^{ads} = k_{OH}^{0}\exp\left[-\frac{\alpha F}{RT}\left(E - E_{OH}^{0'}\right)\right]\exp\left[(\beta - 1)\, g'_{OH}\theta_{OH}\right] \tag{S-8}$$

$$k_{OH}^{des} = k_{OH}^{0}\exp\left[\frac{(1-\alpha)F}{RT}\left(E - E_{OH}^{0'}\right)\right]\exp\left(\beta g'_{OH}\theta_{OH}\right) \tag{S-9}$$

$$\theta_{\mathrm{OH}} = \frac{\Gamma_{\mathrm{OH}}}{\Gamma_{\mathrm{s}}} \quad \text{(S-10)}$$

where c_{H^+} and c_0 are the concentrations of H^+ at the Pt(111) surface and in the bulk solution, respectively, Γ_{OH} and Γ_{s} are the surface concentrations of hydroxyl adsorbates and adsorption sites, respectively, k^0_{OH} is a standard rate constant, α is an electron-transfer coefficient (= 0.5), E is the potential of the Pt(111) substrate, $E^{0'}_{\mathrm{OH}}$ is the formal potential of the hydroxyl adsorption, β is a symmetry factor (= 0.5), and g'_{OH} represents the strength of $\mathrm{OH_{ads}}$–$\mathrm{OH_{ads}}$ interactions. Both HOR and hydroxyl adsorption were considered to define boundary conditions at the substrate as

$$D_{\mathrm{H}^+}\left(\frac{\partial c_{\mathrm{H}^+}}{\partial z}\right) = 2v_{\mathrm{HOR}} - v_{\mathrm{OH}} \quad \text{(S-11)}$$

$$D_{\mathrm{H}_2}\left(\frac{\partial c_{\mathrm{H}_2}}{\partial z}\right) = -v_{\mathrm{HOR}} \quad \text{(S-12)}$$

$$\left(\frac{\partial \Gamma_{\mathrm{OH}}}{\partial t}\right) = v_{\mathrm{OH}} \quad \text{(S-13)}$$

The diffusion problem was solved to calculate a current response at the tip, i_{T}, as given by

$$i_{\mathrm{T}} = 2\pi F \int_0^a r v_{\mathrm{HER}} \mathrm{d}r \quad \text{(S-14)}$$

The tip current was plotted against the substrate potential to yield an SECM-based voltammogram.

The diffusion problem was solved by using the following dimensionless parameters. Specifically, diffusion equations in dimensionless forms are obtained from eqs S-1 and S-2 as

$$\frac{\partial C_{\mathrm{H}^+}}{\partial \tau} = \left(\frac{\partial^2 C_{\mathrm{H}^+}}{\partial R^2} + \frac{1}{R}\frac{\partial C_{\mathrm{H}^+}}{\partial R} + \frac{\partial^2 C_{\mathrm{H}^+}}{\partial Z^2}\right) \quad \text{(S-15)}$$

$$\frac{\partial C_{\mathrm{H_2}}}{\partial \tau} = \gamma \left(\frac{\partial^2 C_{\mathrm{H_2}}}{\partial R^2} + \frac{1}{R} \frac{\partial C_{\mathrm{H_2}}}{\partial R} + \frac{\partial^2 C_{\mathrm{H_2}}}{\partial Z^2} \right) \tag{S-16}$$

with

$$C_{\mathrm{H^+}} = \frac{c_{\mathrm{H^+}}}{c_0} \tag{S-17}$$

$$C_{\mathrm{H_2}} = \frac{c_{\mathrm{H_2}}}{c_0} \tag{S-18}$$

$$R = \frac{r}{a} \tag{S-19}$$

$$Z = \frac{z}{a} \tag{S-20}$$

$$\tau = \frac{D_{\mathrm{H^+}} t}{a^2} \tag{S-21}$$

$$\gamma = \frac{D_{\mathrm{H_2}}}{D_{\mathrm{H^+}}} \tag{S-22}$$

In addition, the potential sweep rate, v, was represented by a dimensionless parameter, σ, as

$$\sigma = \frac{a^2 v F}{D_{\mathrm{H^+}} R T} \tag{S-23}$$

In this work, we considered a = 12.5 μm, v = 0.1 V/s, and $D_{\mathrm{H^+}}$ = 7.8 × 10^{-5} $\mathrm{cm^2/s}$ in eq S-23 to yield σ = 7.7 × 10^{-2}. The normalized surface concentration of adsorption sites, κ, was given by

$$\kappa = \frac{\Gamma_{\mathrm{s}}}{c_0} \sqrt{\frac{F v}{R T D_{\mathrm{H^+}}}} \tag{S-24}$$

We employed Γ_{s} = 0.83 $\mathrm{nmol/cm^2}$, which is the one-third of the surface concentration of Pt(111) atoms (Γ_{Pt} = 2.5 $\mathrm{nmol/cm^2}$) to represent that one $\mathrm{OH_{ads}}$ is formed on one of three Pt atoms as predicted by a

DFT model.[S-5] We obtained $\kappa = 5.5 \times 10^{-1}$ by using c_0 = 1 mM, which is too low to saturate the solution with H_2 as generated at the tip.

Boundary conditions (eqs S-19–S-23) were given by using dimensionless rates. Specifically, eqs S-19 and S-20 were normalized to yield tip boundary conditions as

$$\left(\frac{\partial C_{\mathrm{H}^+}}{\partial Z}\right) = -2V_{\mathrm{HER}}\sqrt{\sigma} \quad \text{(S-25)}$$

$$\gamma\left(\frac{\partial C_{\mathrm{H}_2}}{\partial Z}\right) = V_{\mathrm{HER}}\sqrt{\sigma} \quad \text{(S-26)}$$

with

$$V_{\mathrm{HER}} = \lambda_{\mathrm{HER}}\left(C_{\mathrm{H}^+}\right)^2 \quad \text{(S-27)}$$

$$\lambda_{\mathrm{HER}} = k_{\mathrm{HER}}c_0\sqrt{\frac{RT}{F\nu D_{\mathrm{H}^+}}} \quad \text{(S-28)}$$

Substrate boundary conditions (eqs S-21–S-23) were also normalized to yield

$$\gamma\left(\frac{\partial C_{\mathrm{H}_2}}{\partial Z}\right) = -V_{\mathrm{HOR}}\sqrt{\sigma} \quad \text{(S-29)}$$

$$\left(\frac{\partial C_{\mathrm{H}^+}}{\partial Z}\right) = \sqrt{\sigma}\left(2V_{\mathrm{HOR}} - \kappa V_{\mathrm{OH}}\right) \quad \text{(S-30)}$$

$$\left(\frac{\partial \theta_{\mathrm{OH}}}{\partial \tau}\right) = \sigma V_{\mathrm{OH}} \quad \text{(S-31)}$$

with

$$V_{\mathrm{HOR}} = \lambda_{\mathrm{HOR}}C_{\mathrm{H}_2} \quad \text{(S-32)}$$

$$\lambda_{\mathrm{HOR}} = k_{\mathrm{HOR}}\sqrt{\frac{RT}{F\nu D_{\mathrm{H}^+}}} \quad \text{(S-33)}$$

$$V_{OH}=\lambda_{OH}\left\{C_{H^+}\theta_{OH}\left(\theta_s\right)^{-\alpha}\left(\theta_g\right)^{\beta-1}-\left(1-\theta_{OH}\right)\left(\theta_s\right)^{(1-\alpha)}\left(\theta_g\right)^{\beta}\right\} \quad \text{(S-34)}$$

$$\lambda_{OH}=k^0_{OH}\frac{RT}{Fv} \quad \text{(S-35)}$$

$$\theta_{OH}=\frac{\Gamma_{OH}}{\Gamma_s} \quad \text{(S-36)}$$

$$\theta_s=k^0_{OH}\exp\left[\frac{F}{RT}\left(E-E^{0'}_{OH}\right)\right] \quad \text{(S-37)}$$

$$\theta_g=\exp\left(g'_{OH}\theta_{OH}\right) \quad \text{(S-38)}$$

The diffusion problem based on eqs S-25 and S-26 was first solved under steady states by using COMSOL Multiphysics to yield the initial condition that corresponds to diffusion-limited HER and HOR at the tip and substrate, respectively. The initial substrate potential was negative enough to prevent the hydroxyl adsorption. The time-dependent diffusion problem based on eqs S-25 and S-26 was solved to calculate the normalized tip current during the cycle of the substrate potential.

We also performed the microkinetic analysis of substrate voltammograms in 0.1 M $HClO_4$ (Figure 3A) as well as in 0.5 mM $HClO_4$ and 0.1 M $KClO_4$ (Figure 4B). Fast hydroxyl adsorption (eq 2) was modeled by the Nernst equation based on eq S-7 with v_{OH} = 0 to yield

$$E=E^{0'}_{OH}+\frac{RT}{F}\ln\frac{\theta_{OH}}{1-\theta_{OH}}-\frac{RT}{F}g'_{OH}\theta_{OH} \quad \text{(S-39)}$$

The corresponding substrate current, i_S, was given by

$$i_S=\Gamma_s FA\frac{\partial\theta_{OH}}{\partial t}=\frac{\Gamma_{Pt}FAv}{3}\frac{\partial\theta_{OH}}{\partial E} \quad \text{(S-40)}$$

where A is the electrode surface area, v is the potential scan rate, and $\Gamma_s = \Gamma_{Pt}/3$ as predicted by a DFT model.[S-5] Good fits were obtained at the negative side of sharp butterfly peaks (red dots in Figures 3A and 4B).

The reversible voltammogram of hydrogen adsorption was also fitted by using the corresponding Nernst equation as[S-4]

$$E = E_{\mathrm{V}}^{0'} - \frac{RT}{F}\ln\frac{\theta_{\mathrm{H}}}{1-\theta_{\mathrm{H}}} + \frac{RT}{F}g_{\mathrm{H}}^{'}\theta_{\mathrm{H}} \tag{S-41}$$

with

$$\theta_{\mathrm{H}} = \frac{\Gamma_{\mathrm{H}}}{\Gamma_{\mathrm{Pt}}} \tag{S-42}$$

where $E_{\mathrm{V}}^{0'}$ is the formal potential of the Volmer reaction, Γ_{H} is the surface concentration of the hydrogen adsorbate, and $g_{\mathrm{H}}^{'}$ represents the strength of H_{ads}–H_{ads} interactions. In eq S-42, Γ_{Pt} corresponds to the surface concentration of adsorption sites for H_{ads}. The substrate current was given by

$$i_{\mathrm{S}} = \Gamma_{\mathrm{Pt}}FA\frac{\partial\theta_{\mathrm{H}}}{\partial t} = \Gamma_{\mathrm{Pt}}FAv\frac{\partial\theta_{\mathrm{H}}}{\partial E} \tag{S-43}$$

The calculated current (blue dots in Figure 3A) agreed well with the experimental one.

REFERENCES

(S-1) Fan, F.-R. F.; Demaille, C., The Preparation of Tips for Scanning Electrochemical Microscopy. In *Scanning Electrochemical Microscopy*, Bard, A. J.; Mirkin, M. V., Eds. Marcel Dekker: New York, 2001; pp 75–110.

(S-2) Lefrou, C. A Unified New Analytical Approximation for Positive Feedback Currents with a Microdisk SECM Tip. *J. Electroanal. Chem.* **2006**, *592*, 103–112.

(S-3) Kurapati, N.; Janda, D. C.; Balla, R. J.; Huang, S.-H.; Leonard, K. C.; Amemiya, S. Nanogap-Resolved Adsorption-Coupled Electron Transfer by Scanning Electrochemical Microscopy: Implications for Electrocatalysis. *Anal. Chem.* **2022**, *94*, 17956–17963.

(S-4) Janda, D. C.; Adak, A.; Sivakumar, B.; Amemiya, S. Volcano Plots and Voltammetric Simulation of Electrocatalytic Hydrogen Intermediates at Pt (111) for Scanning Electrochemical Microscopy. *J. Electrochem. Soc.* **2025**, *172*, 066505.

(S-5) Bondarenko, A. S.; Stephens, I. E. L.; Hansen, H. A.; Pérez-Alonso, F. J.; Tripkovic, V.; Johansson, T. P.; Rossmeisl, J.; Nørskov, J. K.; Chorkendorff, I. The Pt(111)/Electrolyte Interface under Oxygen Reduction Reaction Conditions: An Electrochemical Impedance Spectroscopy Study. *Langmuir* **2011**, *27*, 2058–2066.
